# Profiling of Fatty Acids Composition in Suet Oil Based on GC–EI-qMS and Chemometrics Analysis

**DOI:** 10.3390/ijms16022864

**Published:** 2015-01-28

**Authors:** Jun Jiang, Xiaobin Jia

**Affiliations:** 1 Affiliated Hospital on Integration of Chinese and Western Medicine, Nanjing University of Chinese Medicine, Xianlin Avenue 138#, Xianlin University City, Nanjing 210023, China; E-Mail: xuyan9323@126.com; 2 Key Laboratory of New Drug Delivery System of Chinese Meteria Medica, Jiangsu Provincial Academy of Chinese Medicine, 100# Shizi Road, Nanjing 210028, China

**Keywords:** suet oil (SO), composition profiles, fatty acids (FAs), GC–EI-qMS, chemometrics analysis

## Abstract

Fatty acid (FA) composition of suet oil (SO) was measured by precolumn methylesterification (PME) optimized using a Box–Behnken design (BBD) and gas chromatography/electron ionization-quadrupole mass spectrometry (GC–EI-qMS). A spectral library (NIST 08) and standard compounds were used to identify FAs in SO representing 90.89% of the total peak area. The ten most abundant FAs were derivatized into FA methyl esters (FAMEs) and quantified by GC–EI-qMS; the correlation coefficient of each FAME was 0.999 and the lowest concentration quantified was 0.01 μg/mL. The range of recovery of the FAMEs was 82.1%–98.7% (relative standard deviation 2.2%–6.8%). The limits of quantification (LOQ) were 1.25–5.95 μg/L. The number of carbon atoms in the FAs identified ranged from 12 to 20; hexadecanoic and octadecanoic acids were the most abundant. Eighteen samples of SO purchased from Qinghai, Anhui and Jiangsu provinces of China were categorized into three groups by principal component analysis (PCA) according to the contents of the most abundant FAs. The results showed SOs samples were rich in FAs with significantly different profiles from different origins. The method described here can be used for quality control and SO differentiation on the basis of the FA profile.

## 1. Introduction

Suet oil (SO), a fatty oil obtained from the domestic goat (*Capra hircus* Linnaeus) or sheep (*Ovis aries* Linnaeus), has been used in the food industry [1] and the medicine industry [2]. SO is rich in unsaturated and saturated fatty acids (FAs) [3], which are involved in a number of important physiological processes. They provide energy to the cell and act as substrates in the synthesis of fats, lipoproteins, liposaccharides and eicosanoids [4]. Furthermore, SO can be used as an excipient for enhancing the efficacy of traditional Chinese medicines such as Epimedium (Berberidaceae). It was hypothesized that the beneficial effects of Epimedium could be attributed to promotion of the intestinal absorption of drugs by the formation of micelles owing to the action of its FA ingredients [5]. The quality of SO can affect safety and efficacy for clinical patients. There has been little research on the FA composition of SO, however, and there are quality control difficulties in the production of SO. It is important to establish qualitative and quantitative analytical methodology for determining the FA composition of SO.

To date, the methods used for separation and measurement of FAs are mainly chromatographic, including thin-layer chromatography [6], high-performance liquid chromatography [7,8], gas chromatography [9,10,11], supercritical fluid chromatography [12] and liquid chromatography with tandem mass spectrometry [13,14,15,16]. These methods cannot identify major chemical components rapidly and accurately. However, the gas chromatography/electron ionization-quadropole mass spectrometry (GC–EI-qMS) [17,18,19,20] technique coupled with the use of a professional database (NIST 08) can identify many compounds directly and accurately according to their fragment ions and abundance ratio [21,22,23]. In addition, GC–EI-qMS used in the selective ion monitoring (SIM) can identify target compounds rapidly and accurately despite interference from impurities [24], which is especially useful for the analysis of a lipid-based matrix, including SO.

FAs need to be derivatized before they can be analyzed by GC–MS because they have boiling points, which make gasification difficult. In many precolumn derivative methods [25], FAs are normally precolumn methylesterified (PME) into FA methyl esters (FAMEs) [26]. To ensure optimum conditions for methylesterification, the influence of important experimental parameters affecting the efficiency of methylesterification, including methyl reagent volume, temperature and time, were investigated using a Box–Behnken design (BBD) [27,28]. During optimization, the total peak area of the identified FAs was used to select the best conditions. This study developed and validated a method for the qualitative and quantitative profiling of the FA content in SOs for the first time.

SOs have been used widely in medicinal and culinary areas, but their authentication and standardization have encountered some problems owing to deliberate contamination with other animal or vegetable oils. It is difficult to identify the origins and species of SO accurately on the basis of appearance and morphology. Furthermore, SOs from different species or from different regions are not of uniform composition. In this study, a total of 18 batches of SO collected from three provinces in China were analyzed by GC–EI-qMS to determine their FA compositions and principal component analysis (PCA) was used to evaluate and classify these samples.

## 2. Results and Discussion

### 2.1. Optimal Results and Statistical Analysis of Precolumn Methylesterified (PME)

By retaining only the factors statistically significantly different at *p* ≤ 0.05, the following final equation in terms of uncoded factors was obtained:

Total peak area = +3.954 × 10^9^ + 9.987 × 10^8^A + 1.196 × 10^9^B + 9.163 × 10^8^C + 1.099 × 10^9^AB + 8.977 × 10^8^AC + 9.306 × 10^8^B − 1.596 × 10^9^A^2^ − 1.232 × 10^9^B^2^ − 1.452 × 10^9^C^2^.

**Figure 1:**
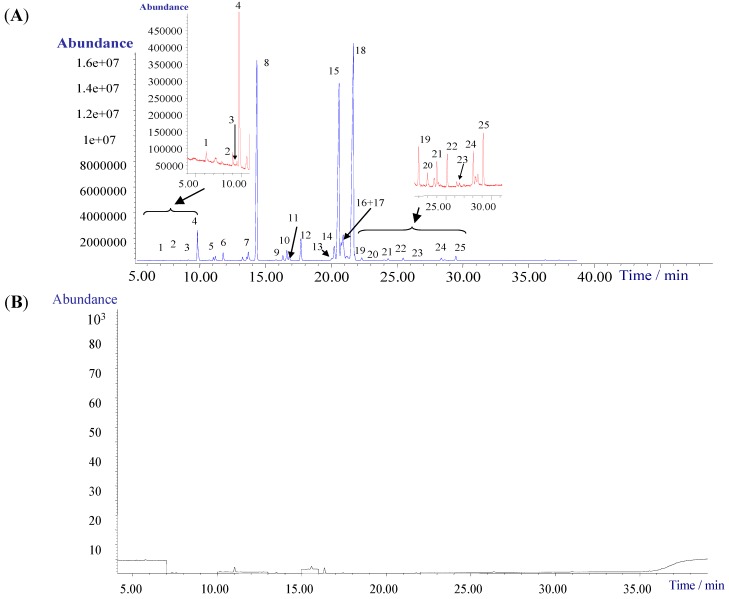

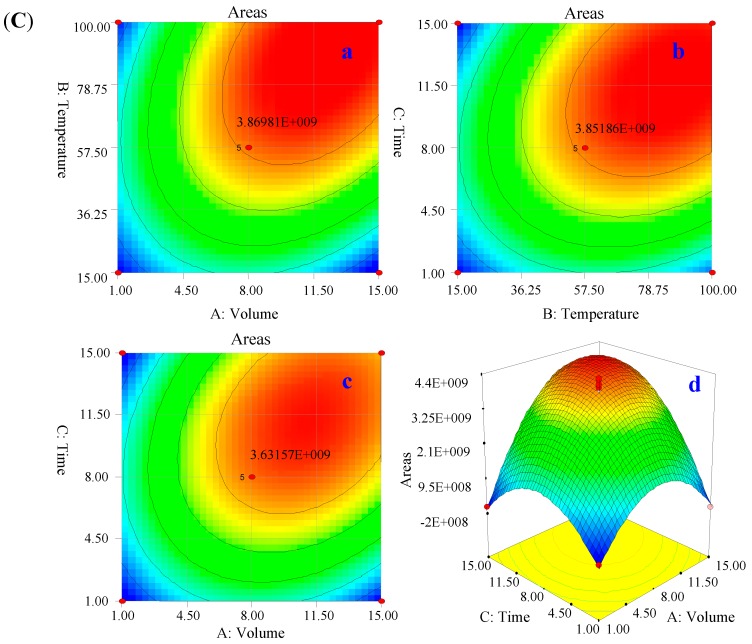
Optimization of precolumn methylesterified (PME) by Box–Behnken design (BBD)/GC–EI-qMS. (**A**) GC–EI-qMS chromatogram of the 25 fatty acid methyl esters (FAMEs) in suet oil (SO) sample under total ion chromatogram (TIC) mode. (**1**) Dodecanoic acid, methyl ester (DODME), (**2**) Methyl myristoleate, methyl ester, (**3**) Methyl 12-methyl-tridecanoate, methyl ester, (**4**) Tridecanoic acid, 12-methyl-, methyl ester, (**5**) Methyl tetradecanoate, methyl ester (MTEME), (**6**) Pentadecanoic acid, methyl ester (PENME), (**7**) (*Z*)-9-Hexadecenoic acid, methyl ester (9-HEME), (**8**) Hexadecanoic acid, methyl ester (HEXME), (**9**) Methyl 15-methylhexadecanoate, methyl ester, (**10**) *cis*-10-Heptadecenoic acid, methyl ester, (**11**) Heptadecanoic acid, methyl ester (HEPME), (**12**) (*Z*,*Z*)-9,12-Octadecadienoic acid, methyl ester (9,12-OCME), (**13**) Methyl 9-*cis*,11-*trans*-octadecadienoate methyl ester, (**14**) Methyl 10-*trans*,12-*cis*-octadecadienoate, (**15**) 9-Octadecenoic acid (*E*)-, methyl ester (9-OCME), (**16**) 9-Octadecenoic acid (*Z*)-, methyl ester, (**17**) 11-Octadecenoic acid, methyl ester, (**18**) Octadecanoic acid, methyl ester (OCTME), (**19**) *cis*-10-Nonadecenoic acid, methyl ester, (**20**) 10-Nonadecenoic acid, methyl ester, (**21**) Cyclopropaneoctanoic acid, 2-octyl-, methyl ester, (**22**) Nonadecanoic acid, methyl ester, (**23**) Methyl 8,11,14-eicosatrienoate, methyl ester, (**24**) *cis*-11-Eicosenoic acid, methyl ester, (**25**) Eicosanoic acid, methyl ester (EICME); (**B**) GC–EI-qMS chromatogram of representative blank samples under TIC mode; (**C**) Response surface plots (3-D) and contour (2-D) showing the total peaks areas with different methyl esterified condition. (**a**) 2-D panel of temperature-volum, (**b**) 2-D panel of time-temperature, (**c**) 2-D panel of time-volum, (**d**) 3-D response surface plots.

In all, 25 FA species can be identified from the chromatogram shown in Figure 1A. Comprehensive test results for response surface plots (3D) and contour plots (2D) show the total peak area was a maximum when the methylesterification conditions were: reagent volume 10 mL; temperature 60 °C; and time 10 min (Figure 1C).

### 2.2. Fatty Acids (FAs) Composition in Suet Oil (SO)

Identification of FAs was achieved by comparing molecular mass, ion fragments and abundance ratios in the NIST 08 spectral library. A typical total ion chromatogram obtained for SO samples is shown in Figure 1A. 

The FAs in SO were investigated using optimized PME: 10 mL of BF_3_–MeOH (14%, *v*/*v*), 60 °C and 10 min. The PME/GC–EI-qMS analysis of SO led to the identification of 25 different FAs (Table 1): including saturated FAs (dodecanoic acid, 12-methyl-tridecanoate, tridecanoic acid, 12-methyl-tetradecanoate, pentadecanoic acid, hexadecanoic acid, heptadecanoic acid, octadecanoic acid, cyclopropaneoctanoic acid, nonadecanoic acid, eicosanoic acid, *cis*-11-eicosenoic acid and 8,11,14-eicosatrienoate) and unsaturated FAs (myristoleate, 9-hexadecanoic acid, *cis*-10-heptadecanoic acid, (*Z*,*Z*)-9,12-octadecadienoic acid, 10-nonadecanoic acid, 10-*trans*,12-*cis*-octadecadienoate, (*E*)-9-octadecanoic acid, (*Z*)-9-octadecanoic acid, 11-octadecanoic acid, *cis*-10-nonadecanoic acid and 9-*cis*,11-*trans*-octadecadienoate).

In all, 25 FAs were identified (match > 90%). Hexadecanoic acid, octadecanoic acid and (*E*)-9-octadecanoic acid were the three most abundant and occupied 16.46%, 37.96% and 19.47% of the total peak area, respectively (Table 1).

### 2.3. Validation of Quantitative Analysis

Methylester derivatives of the ten most abundant FAs were purchased for use as standards. These FAs in the SO samples were quantified by derivatization into FAMEs, which were analyzed by GC–EI-qMS without significant matrix interference. Four fragment ions were monitored in the SIM mode for each compound. The best characteristic ion in the spectrum was selected for quantification of each FAME and the other three were used for confirmation.

**Figure 2:**
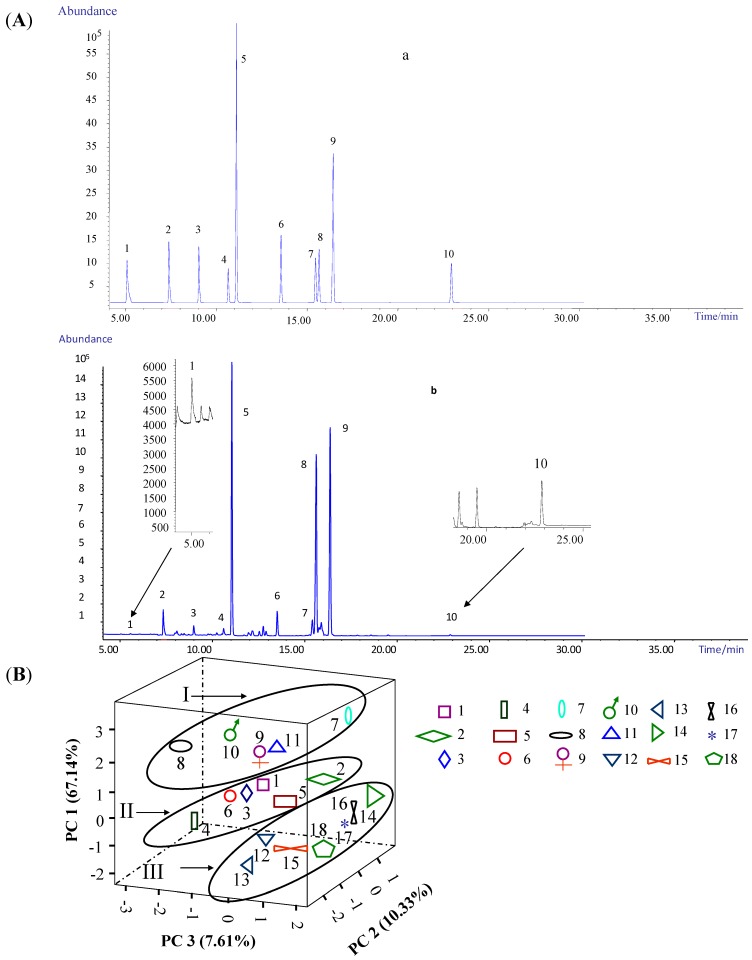
GC–EI-qMS chromatograms of the ten FAMEs standard mixture and sample under SIM mode and principal component analysis (PCA) of 18 SO samples. (**A**) (**a**) mixed standard solution (the concentration of (**1**–**10**) was 10.8, 47.6, 31.6, 13.0, 11.2, 12.8, 10.0, 20.0, 20.0 and 20.0 μg/mL, respectively), (**b**) SO sample solution. (**1**) DODME, (**2**) MTEME, (**3**) PENME, (**4**) 9-HEXME, (**5**) HEXME, (**6**) HEPME, (**7**) 9,12-OCME, (**8**) 9-OCME, (**9**) OCTME, (**10**) EICME; (**B**) The 3D scatter plot obtained by PCA of 18 SO samples.

The validity of the method was investigated by examination of the linearity, recovery, and limit of quantification (LOQ) for all FAMEs in this study. The ranges of concentration, regression equations, *r*^2^ (coefficient of determination), recovery, relative standard deviation (RSD) and LOQ for the target FAMEs are given in Table 2. Most of the FAMEs had good linearity (*r*^2^ > 0.999); more importantly, the results showed a stabilized recovery of ten FAMEs in the range 82.1%–98.7% with optimized PME parameters (Table 2, Figure S1) and the LOQ values of these FAMEs ranged from 1.25 to 5.95 μg/L. These results indicated LOQ was sufficiently low to meet the requirements of determination of the FA composition of SOs. A chromatogram of these ten FAMEs in SOs obtained in the SIM mode is shown in Figure 2A. 

**Table 1:** Fatty acids identified in SO sample using precolumn esterified/GC–EI-qMS.

No.	RT (min)	Compounds Name	CAS No.	*M*w ^a^	Formula	Match (%)	RC ^b^ (%)
1	6.142	Dodecanoic acid, methyl ester	000111-82-0	214	C_13_H_26_O_2_	98	0.11
2	9.016	Methyl myristoleate	056219-06-8	240	C_15_H_28_O_2_	96	0.19
3	9.504	Methyl 12-methyl-tridecanoate	1000336-46-9	242	C_15_H_30_O_2_	98	0.087
4	9.739	Tridecanoic acid, 12-methyl-, methyl ester	005129-58-8	242	C_15_H_30_O_2_	94	0.25
5	11.071	Methyl tetradecanoate	000124-10-7	242	C_15_H_30_O_2_	98	2.52
6	11.663	Pentadecanoic acid, methyl ester	007132-64-1	256	C_16_H_32_O_2_	99	0.29
7	13.614	9-Hexadecenoic acid, methyl ester, (*Z*)-	001120-25-8	268	C_17_H_32_O_2_	99	2.51
8	14.311	Hexadecanoic acid, methyl ester	000112-39-0	270	C_17_H_34_O_2_	98	16.46
9	16.253	Methyl 15-methylhexadecanoate	1000336-34-2	284	C_18_H_36_O_2_	99	0.64
10	16.531	*cis*-10-Heptadecenoic acid, methyl ester	1000333-62-1	282	C_18_H_34_O_2_	99	0.98
11	16.723	Heptadecanoic acid, methyl ester	001731-92-6	284	C_18_H_36_O_2_	99	1.06
12	17.507	9,12-Octadecadienoic acid (*Z*,*Z*)-, methyl ester	000112-63-0	294	C_19_H_34_O_2_	99	1.61
13	19.492	Methyl 9-*cis*,11-*trans*-octadecadienoate	1000336-44-0	294	C_19_H_34_O_2_	95	1.29
14	19.919	Methyl 10-*trans*,12-*cis*-octadecadienoate	1000336-44-2	294	C_19_H_34_O_2_	96	0.10
15	20.119	9-Octadecenoic acid (*E*)-, methyl ester	001937-62-8	296	C_19_H_36_O_2_	99	37.96
16	20.546	9-Octadecenoic acid (*Z*)-, methyl ester	000112-62-9	296	C_19_H_36_O_2_	99	1.97
17	20.955	11-Octadecenoic acid, methyl ester	052380-33-3	296	C_19_H_36_O_2_	99	2.05
18	21.992	Octadecanoic acid, methyl ester	000112-61-8	298	C_19_H_38_O_2_	99	19.47
19	22.166	*cis*-10-Nonadecenoic acid, methyl ester	1000333-64-4	310	C_20_H_38_O_2_	98	0.065
20	23.316	10-Nonadecenoic acid, methyl ester	056599-83-8	310	C_20_H_38_O_2_	93	0.24
21	24.308	Cyclopropaneoctanoic acid, 2-octyl-, methyl ester	3971-54-8	310	C_20_H_38_O_2_	99	0.42
22	25.287	Nonadecanoic acid, methyl ester	001731-94-8	312	C_20_H_40_O_2_	99	0.26
23	26.829	Methyl 8,11,14-eicosatrienoate	1000336-38-1	320	C_21_H_36_O_2_	97	0.034
24	28.262	*cis*-11-Eicosenoic acid, methyl ester	1000333-63-8	324	C_21_H_40_O_2_	99	0.12
25	29.653	Eicosanoic acid, methyl ester	001120-28-1	326	C_21_H_42_O_2_	99	0.20

^a^ *M*w = Molecular Weight (nominal values); ^b^ RC (%) = The relative content of total peak areas, the sum of the RC was 90.89%, the other 9.11% may contain inorganic elements, glycerin and something else.

**Table 2:** The linear regression equations, the correlation coefficient (*r*), limit of quantification (LOQ), recoveries of 10 fatty acids under GC–EI-qMS selective ion monitoring (SIM) conditions.

NO. of Identified Fatty Acids	Compounds	Linear Regression Equations	Coefficient of Determination/*r*^2^	Linear Range μg/mL	Qualitative/Quantitative Ions	Abundance Ratio (%)	0.5 Times Spiked (*n = *3)		1.0 Times Spiked (*n = *3)		2.0 Times Spiked (*n = *3)		LOQ (μg/mL, ×10^−3^)
							Recovery (%)	RSDs (%)	Recovery (%)	RSDs (%)	Recovery (%)	RSDs (%)	
1	DODME	*Y* = 1.56 × 10^4^ *X* − 1.79 × 10^3^	0.999	0.010–10.0	74 *:28:87:214	100:8:64:6	85.3	4.3	92.4	4.8	93.2	2.2	1.25
5	MTEME	*Y* = 1.54 × 10^4^ *X* − 1.48 × 10^3^	0.999	0.013–12.8	74 *:87:143:199	100:68:24:16	95.2	4.1	97.2	3.3	98.7	4.5	1.60
6	PENME	*Y* = 1.86 × 10^4^ *X* − 4.74 × 10^3^	0.999	0.011–12.0	74 *:87:143:213	100:68:20:18	91.3	6.2	92.8	4.5	95.8	4.2	1.40
7	9-HEXME	*Y* = 3.10 × 10^3^ *X* − 1.13 × 10^3^	0.999	0.020–20.0	55 *:74:87:236	100:68:50:23	83.6	4.8	94.6	3.5	96.6	2.4	2.50
8	HEXME	*Y* = 2.62 × 10^4^ *X* − 1.579 × 10^4^	0.999	0.048–50.0	74 *:87:143:227	100:70:20:14	87.5	5.2	95.4	5.4	96.9	3.8	5.95
11	HEPME	*Y* = 2.93 × 10^4^ *X* − 6.54 × 10^3^	0.999	0.013–13.0	74 *:87:143:241	100:70:22:15	87.6	6.8	90.8	5.0	92.7	4.1	1.63
12	9,12-OCME	*Y* = 7.71 × 10^3^ *X* − 1.15 × 10^2^	0.999	0.020–20.0	67 *:81:95:294	100:92:66:16	91.7	4.4	91.7	2.8	93.9	3.6	2.50
15	9-OCME	*Y* = 5.02 × 10^3^ *X* − 3.62 × 10^2^	0.999	0.020–20.0	55 *:41:81:222	100:62:40:24	84.8	5.1	88.4	4.2	97.2	4.4	2.50
18	OCTME	*Y* = 3.31 × 10^4^ *X* − 1.24 × 10^4^	0.999	0.030–32.0	74 *:87:143:255	100:72:23:14	85.5	4.3	86.7	5.2	88.8	5.1	3.75
25	EICME	*Y* = 3.18 × 10^4^ *X* − 4.78 × 10^3^	0.999	0.010–10.8	74 *:87:143:255	100:76:26:18	82.1	3.9	90.1	4.7	89.4	3.7	1.25

* Quantitative ion, LOQ was calculated as 10 times of the signal to noise ratio (10 S/N). 0.5, 1.0 and 2.0 times “Spiked” means the added standards amount was 0.5, 1.0 and 2.0 times of the initial content of samples.

### 2.4. Quantitative Results

The molecular species detected after methylesterification were FAMEs rather than FAs. Therefore, in order to quantify the FAs, the contents of FAMEs were converted into FAs by multiplication with the corresponding coefficient as follows:

*W*_FAs_ (%) = *A* × *W*_FAMEs_ (%) (1)

where *W *is concentration and *A* is the molecular mass of the FAMEs*/*the molecular mass of the FAs.

The results for ten FAs in 18 batches of SO are given in Table 3. The contents of hexadecanoic acid and octadecanoic acid were 3.79%–13.22% and 3.41%–18.11%, respectively. The contents of (*E*)-9-octadecanoic acid in SO batches 3–6, 8, 12–15 and 18 were 4.48%–6.28%, second only to hexadecanoic acid and octadecanoic acid, whereas no other FA was detected. On the basis of the quantitative results for 18 batches of SO, the content of octadecanoic acid was greatest, followed by hexadecanoic acid, (*E*)-9-octadecanoic acid and tetradecanoate in that order.

### 2.5. Principal Component Analysis (PCA) of the SO Samples

In order to evaluate the variation between batches of SO, PCA was applied on the basis of the contents of the ten most abundant FAs. The first three principal components (PC1, PC2 and PC3) with >85% of the whole variance were extracted for analysis. PC1, PC2 and PC3 accounted for 67.14%, 10.33% and 7.61% of the total variance, respectively (Table S1). The remaining principal components had only a minor effect on the model and were discarded. The component loading matrix is given in Table S2 and Table S3. According to the loadings, PC1 had a good correlation with each of the ten FA compounds. The results mentioned above suggested that most of the compounds contributed to the classification of the samples. The scatter plots are shown in Figure 2B, where each sample is represented as one marker.

The dots of 18 samples were classified into group I, group II or group III in accord with their origin. Dots in groups II and III were relatively close to each other, indicating a close relationship among the six batches from Anhui and the seven batches from Jiangsu. The dots in group I were quite scattered, suggesting diversification of the five batches from Qinghai Province. These observations might be explained as follows. Firstly, the land area of Qinghai Province (722,300 km^2^) is larger compared to Anhui Province (1,396,002 km^2^) and Jiangsu Province (106,700 km^2^), representing a greater area for diversity of the samples. Secondly, Qinghai, Anhui and Jiangsu provinces are considerably different environments with large differences in climate, which influences the differences in FA metabolism in domestic sheep and goats. Thirdly, Anhui and Jiangsu provinces are geographic neighbors, which is reflected in the similarities among samples from these two origins. Finally, the SO samples from Qinghai Province were from sheep, whereas those obtained from Anhui and Jiangsu provinces were from goats.

**Table 3:** Contents of ten FAs in 18 batches of Suet oil (*n* = 3).

FAs Compounds	DODME	MTEME	PENME	9-HEXME	HEXME	HEPME	9,12-OCME	9-OCME	OCTME	EICME
**Coefficient “A” ^a^**	0.9348	0.9414	0.9454	0.9478	0.9482	0.9508	0.9524	0.9527	0.9531	0.9571
**Batch**	**Content (g/100 g, %) ^b^**									
1	0.0240 ± 0.0014	1.1476 ± 0.0014	0.2124 ± 0.0002	0.5716 ± 0.0012	8.9067 ± 0.1483	0.4996 ± 0.0006	0.8667 ± 0.0015	Nd	12.5609 ± 0.1614	0.0933 ± 0.0021
2	0.0284 ± 0.0010	1.0827 ± 0.0012	0.1787 ± 0.0015	0.4596 ± 0.0029	7.6098 ± 0.0040	0.5022 ± 0.0011	1.1867 ± 0.0023	Nd	11.0827 ± 0.0011	0.1040 ± 0.0023
3	0.0284 ± 0.0009	0.8684 ± 0.0010	0.2009 ± 0.0028	0.4044 ± 0.0025	6.2578 ± 0.0157	0.5289 ± 0.0053	1.1218 ± 0.0102	Nd	8.7600 ± 0.3606	0.0942 ± 0.0013
4	0.0196 ± 0.0004	0.4862 ± 0.0015	0.2098 ± 0.0039	0.4204 ± 0.0023	4.4382 ± 0.0011	0.4551 ± 0.0022	0.6587 ± 0.0065	Nd	4.0124 ± 0.0017	0.0471 ± 0.0008
5	0.0231 ± 0.0023	0.7707 ± 0.0083	0.1991 ± 0.0017	0.3191 ± 0.0017	5.8071 ± 0.0045	0.5707 ± 0.0015	0.6604 ± 0.0024	4.5991 ± 0.0053	10.2213 ± 0.0163	0.1467 ± 0.0032
6	0.0418 ± 0.0012	1.1912 ± 0.0034	0.2844 ± 0.0012	1.3378 ± 0.0051	7.5067 ± 0.0074	0.6187 ± 0.0052	0.9227 ± 0.0008	Nd	8.3093 ± 0.0297	0.0978 ± 0.0017
7	0.0400 ± 0.0031	1.9422 ± 0.0068	0.3040 ± 0.0108	1.1564 ± 0.0020	13.2151 ± 0.0241	0.7956 ± 0.0023	1.4640 ± 0.0028	Nd	18.1129 ± 0.0028	0.1662 ± 0.0035
8	0.0328 ± 0.0017	1.1182 ± 0.0037	0.2729 ± 0.0017	0.7662 ± 0.0026	7.9458 ± 0.0026	0.7218 ± 0.0017	0.9048 ± 0.0033	Nd	12.1111 ± 0.0015	0.1582 ± 0.0001
9	0.0400 ± 0.0031	1.4516 ± 0.0012	0.3902 ± 0.0035	0.8649 ± 0.0033	10.8133 ± 0.0034	1.1004 ± 0.0001	1.4924 ± 0.0035	Nd	12.9360 ± 0.0275	0.1111 ± 0.0016
10	0.0280 ± 0.0046	1.0978 ± 0.0045	0.2516 ± 0.0024	0.4222 ± 0.0029	8.264 ± 0.0394	0.6960 ± 0.0027	1.0480 ± 0.0042	Nd	13.0320 ± 0.0337	0.1467 ± 0.0040
11	0.0373 ± 0.0035	1.1991 ± 0.0031	0.4791 ± 0.0033	0.4764 ± 0.0040	10.0676 ± 0.0045	0.9218 ± 0.0017	0.8107 ± 0.0059	Nd	14.2569 ± 0.0029	0.1040 ± 0.0016
12	0.0356 ± 0.0039	1.0196 ± 0.0041	0.2658 ± 0.0039	0.7564 ± 0.0031	6.7449 ± 0.0017	0.4942 ± 0.0033	0.7787 ± 0.0041	6.2764 ± 0.0023	6.0773 ± 0.0036	0.0560 ± 0.0034
13	0.0178 ± 0.0040	0.4080 ± 0.0046	0.1769 ± 0.0034	0.3396 ± 0.0044	3.7902 ± 0.0047	0.3804 ± 0.0018	0.5449 ± 0.0039	5.4489 ± 0.0048	3.4080 ± 0.0051	0.0382 ± 0.0012
14	0.0322 ± 0.0035	1.1831 ± 0.0040	0.2391 ± 0.0045	0.4276 ± 0.0046	8.3760 ± 0.0164	0.5822 ± 0.0029	1.2240 ± 0.0167	5.2880 ± 0.0282	11.2276 ± 0.0060	0.087 ± 0.0045
15	0.0240 ± 0.0023	0.6462 ± 0.0034	0.1458 ± 0.0028	0.4587 ± 0.0013	4.6827 ± 0.0034	0.3671 ± 0.0035	0.6240 ± 0.0293	5.0649 ± 0.0034	6.6560 ± 0.0220	0.0773 ± 0.0038
16	0.0267 ± 0.0029	0.8071 ± 0.0039	0.1636 ± 0.0032	0.3363 ± 0.0032	5.8738 ± 0.0015	0.4747 ± 0.0038	1.0738 ± 0.0028	4.8996 ± 0.0044	9.0898 ± 0.0042	0.0969 ± 0.0023
17	0.0267 ± 0.0034	0.7911 ± 0.0061	0.1564 ± 0.0028	0.3209 ± 0.0078	5.4720 ± 0.0406	0.4329 ± 0.0030	1.0169 ± 0.0033	4.6578 ± 0.0046	8.1529 ± 0.0040	0.0862 ± 0.0039
18	0.0160 ± 0.0051	0.6969 ± 0.0034	0.0907 ± 0.0064	0.3413 ± 0.0031	5.5209 ± 0.0071	0.2480 ± 0.0046	0.5013 ± 0.0034	4.4756 ± 0.0034	7.9644 ± 0.0033	0.0622 ± 0.0042

^a^ A = Molecular Weight _(FAMEs)_/Molecular Weight _(FAs)_; ^b^ W_FAs_ (%) = A × W_FAMEs_ (%); Nd = not detected.

## 3. Experimental Section

### 3.1. Materials

Methyl dodecanoic acid (DODME ≥ 98.0 (purity)), methyl tetradecanoate (MTEME ≥ 99.0), methyl pentadecanoic acid (PENME ≥ 98.0), methyl 9-hexadecenoic acid (*Z*) (9-HEME ≥ 99.0), methyl hexadecanoic acid (HEXME ≥ 99.0), methyl heptadecanoic acid (HEPME ≥ 99.0), 9,12-methyl octadecadienoic acid (*Z*,*Z*) (9,12-OCME ≥ 99.0), methyl 9-octadecenoic acid (*E*) (9-OCME ≥ 99.0), methyl octadecanoic acid (OCTME ≥ 98.0), methyl eicosanoic acid (EICME ≥ 99.0), boron trifluoride-methanol (14%, *v*/*v*), sodium hydroxide (NaOH) and sodium (NaCl) were purchased from Anpel Scientific Instrument Co., Ltd. (Shanghai, China). HPLC grade methanol, and *n-*hexane were obtained from Merck (Darmstadt, Germany).

### 3.2. Sample Material

Eighteen batches of SO samples were purchased from Qinghai (batches 7–11), Jiangsu (batches 12–18) and Anhui (batches 1–6) provinces, China between January and July 2013 (Figure S2). All samples were stored in darkness at temperatures <4 °C. For the blank sample, the *n*-hexane was used instead of SOs.

### 3.3. Box–Behnken Design for Optimization of PME Parameters

The application of an effective PME methodology requires optimization of the main parameters that influence the methylesterification process, including the volume of methyl reagent, temperature and time.

A Box–Behnken Design, a response surface methodology, was used in this study. Design Expert 7.0.0 software was used for analyzing the experimental data. The study type was Response Surface, the initial design was Box–Behnken, the design model was Quadratic and Blocks was No Blocks. A Box–Behnken statistical screening design with three independent variables (*A*, PME volume; *B*, PME temperature; *C*, PME time) was used to optimize the PME process for the qualitative and quantitative analysis. Statistically significant difference was set at *p* ≤ 0.05. The *r*^2^ value of the “Final Equation” > 0.995 indicated derived results were accurate. Data were expressed as mean ± standard deviation (SD) of triplicate determinations. Statistical calculations used Statistical Product and Service Solutions (SPSS) version 16.0 software (SPSS Inc., Chicago, IL, USA). One-way ANOVA was used for evaluating the statistical differences among samples.

### 3.4. PME Procedure

A 0.4 g sample of SO from batch 12 was weighed and placed into a 50-mL conical flask followed by 15 mL NaOH–MeOH (0.5 mol/L) then heated at 60 °C in a waterbath for 20 min until the yellow beads of SO disappeared completely after cooling. The flask contents were subjected to the PME procedure, in which 10 mL of boron trifluoride methanol (BF_3_–MEOH, 14% *v*/*v*) was added to the flask then heated at 60 °C in a waterbath for 10 min. The mixture was cooled and then 10 mL of *n*-hexane and 10 mL of saturated NaCl were added. Samples 1.5 mL of supernatants were injected through a 0.45-µm pore size membrane before GC–EI-qMS qualitative analysis.

### 3.5. Sample Pretreatment for Quantitative Analysis

Eighteen batches of SO were treated as described in section 3.4 above. Sequentially, 25-μL was transferred into a 10-mL volumetric flask followed by addition of *n*-hexane to a final volume of 10 mL and then shaken. After passage through an organic 0.45-µm pore size filter, the treated samples were injected into the GC–EI-qMS for quantitative analysis.

### 3.6. Preparation of Standard Solutions

Stock solutions of the ten FAMEs (DODME, MTEME, PENME, 9-HEXME, HEXME, HEPME, 9,12-OCME, EICME, OCTME and 9-OCME) were prepared in *n*-hexane at concentrations of 20.0, 11.2, 20.0, 12.8, 31.6, 47.6, 10.8, 20.0, 13.0 and 10.0 μg/mL, respectively. Appropriate amounts of the above stock solutions were mixed and diluted into a series of concentrations with *n*-hexane to obtain the working solutions. All solutions were stored at <4 °C.

### 3.7. GC–EI-qMS Analysis Conditions

For separation, detection and identification of FAs, the qualitative and quantitative analyses were made with a GC–EI-qMS instrument (Agilent 7890/5975) coupled to an automatic sampler (Agilent 7693) and an electron impact ionization source (Agilent, Santa Clara, CA, USA). Water was purified by a Milli-Q Plus apparatus (Millipore, Bedford, MA, USA). The H2050R centrifugal apparatus was provided by the Hunan Saite xiangyi centrifuge instrument Co., Ltd. (Xiangya, China).

Analytes were separated using a 30 m × 0.25 mm capillary column (HP-5 ms 0.25 μm film thickness; Agilent Technology, Santa Clara, CA, USA). The primary oven temperature protocol was: 150 °C for 1 min; increased to 200 °C at 5 °C/min; maintained at this temperature for 5 min; increased to 250 °C at a rate of 5 °C /min; maintained at this temperature for 5 min; increased to 300 °C at a rate of 5 °C/min; and maintained at this temperature for 10 min. The injection port temperature was 250 °C. The carrier gas was helium at a constant flow of 1 mL/min. The MS operating conditions in the splitless injection mode were as follows: ion source temperature 280 °C; electron energy 70 eV; emission current 250 μA; injection volume 0.2 μL; and solvent delay 4 min. The SIM mode was used for quantitative determination of FAs.

### 3.8. Method for PCA of Samples

PCA was done with SPSS 16.0 software (SPSS, Chicago, IL, USA) [29]. In this study, the contents of the ten FAs in the 18 SO samples were used as a data matrix with 18 rows and ten columns for PCA analysis after normalization. The first three PCs were extracted, and the scatter plot was obtained by plotting the scores of PC1* vs.* PC2 and PC3.

## 4. Conclusions

The optimal conditions for methylesterification of FAs were obtained by a Box–Behnken Design, which identified 25 kinds of FAs in SO by GC–EI-qMS. In addition, ten FAs in 18 batches of SO were analyzed with good performance with regard to selectivity, recovery, precision and accuracy. Significant differences among origins in FA composition profiles and their contents were revealed. The method described here could be used in quality control and standardization of SOs and their products as well as providing supportive chemical information.

## Supplementary Materials

Supplementary materials can be found at http://www.mdpi.com/1422-0067/16/02/2864/s1.
